# Workplace violence against Chinese licensed doctors: a cross-sectional study

**DOI:** 10.3389/fpubh.2024.1235576

**Published:** 2024-01-31

**Authors:** Wenhao Chen, Jingyu Shi, Jingyi Xu, Yue Wang, Yanbin Wu

**Affiliations:** ^1^School of Health Humanities, Peking University, Beijing, China; ^2^School of Public Health, Peking University, Beijing, China

**Keywords:** workplace violence, licensed doctors, prevention, China, cross-sectional study

## Abstract

**Introduction:**

China has issued and implemented a series of policies aimed at preventing and controlling workplace violence (WPV) against licensed doctors. However, the prevalence of WPV has not been fundamentally curbed. The aim of this study was to present the prevalence of WPV, identify its influencing factors and propose responsive measures.

**Method:**

The online Chinese Physician Practice Survey was conducted with purposive sampling method among licensed doctors in China from January 2022 to June 2022. Data covered licensed doctors’ sociodemographic characteristics, occupational characteristics, prevalence of WPV, and perception of effective countermeasures.

**Results:**

A total of 74,305 licensed doctors participated in this study. A total of 44.88% of respondents had experienced WPV, among them, either physical violence only (1.06%) or non-physical violence only (89.91%) or encountered both of them (9.03%). Age, gender, marital status, education level, professional title and registration type were all associated with WPV, being younger, non-married, more educated, and higher professional title are all risk factors for WPV. Male (OR = 1.396, 95CI%: 1.355 to 1.439), clinicians (OR = 1.342,95%CI: 1.177 to 1.529), who were single (OR = 1.174, 95%CI: 1.111 to 1.241), with master’s degree (OR = 2.021, 95%CI: 1.739 to 2.349) and professional title were subsenior (OR = 1.194, 95%CI: 1.125 to 1.267) were most likely to occur WPV. WPV occurred mostly in provincial capitals, public hospitals, primary and community hospitals, and departments of internal medicine, surgery, pediatrics, emergency medicine and mental health. Overall, 44.24% of doctors perceived that strengthening crackdowns on criminal behaviors was the most effective measure to prevent WPV against healthcare staff.

**Conclusion:**

The frequency of WPV decreased after the implementation of relevant laws and policies. Future efforts should be made to strengthen crackdowns on illegal and criminal activities and to issue specific legal provisions on the prevention and control of WPV against doctors.

## 1 Introduction

Workplace violence (WPV) refers to a situation in which an individual is harassed, threatened, or attacked at work (1). It can be classified into two categories: physical violence, such as physical attacks and killings, and non-physical violence, such as verbal abuse and sexual harassment (2). WPV results in negative consequences, including physical injuries, mental health issues, decreased quality of patient care, and medical errors that may worsen the doctor–patient relationship (3).

In recent years, WPV against licensed doctors has become a serious social problem in China (4). Before 2015, the *Public Security Administration Punishments Law* was the only law that regulated WPV against doctors in China (5). As an increasing number of WPV cases were reported in healthcare settings, China enacted legislation against violence toward doctors (6). In 2015, *Criminal Law* added the disruption of medical services as a criminal offense and set a punishment of 3–7 years’ imprisonment for violations (7). Therefore, the incidence rate of WPV in hospitals gradually decreased in the following years (4). Later, in 2018, the Chinese State Council approved the *Regulation on the Prevention and Handling of Medical Disputes*, which explicitly stipulates that individuals who disturb medical services, violate public safety, or cause substantial harm shall hold civil, administrative, and criminal liabilities accordingly (8). On June 1, 2020, China’s first comprehensive legislation on medical services, the *Law on the Promotion of Basic Medical and Health Care*, came into effect, further strengthening legal protection for doctors (9). On August 20, 2021, the issuance of the *Law on Licensed Doctors* marked the first legal document that specifically protected licensed doctors’ rights (10).

In addition to legislation, a set of policies have been introduced to control and prevent WPV against doctors. In 2013, the *Guidance on Strengthening the Construction of Hospital Safety Prevention Systems* suggested that healthcare institutions should build a three-dimensional defense system that combines civil, physical, and technological defense components (11). In 2014, several governmental departments issued the *Opinions on Punishment of Illegal Crimes in Medical Services to Maintain Health Institutions’ Normal Order*, which noted six types of criminal behaviors for the first time (12). On September 5, 2014, the *Notification on Strengthening Safe and Secure Hospital Establishment Activities to Strengthen the Crackdown on Medical Crimes and Maintain Normal Medical Order* introduced various measures to prevent, control and intervene in WPV, including intensifying crackdowns on criminal behaviors, strengthening legal and security education for doctors, and truthfully reporting when patients initiated WPV incidents (13). After 2015, China further introduced a series of policies, including *the Opinions on Fully Fulfilling the Procuratorate’s Functions to Provide Powerful Judicial Safeguards for Advancing the Construction of a Healthy China* (14), *Opinions on Strictly Preventing and Controlling Medical-related Illegal and Criminal Activities to Maintain Normal Medical Order* (15), and *Guiding Opinions on Promoting Safe Order Management in Hospitals* (16). However, these laws and policies are, in many cases, scattered, inconsistent, and infeasible, resulting in a high rate of WPV against doctors.

There have been relatively few previous studies on WPV against doctors since the introduction of these legislation, regulations, and policies. In addition, previous studies on WPV against doctors in China have usually concentrated on reported violent incidents, which were attention-grabbing but often underreported (17, 18). Furthermore, these studies often involved doctors in certain departments, hospitals, or provinces and failed to be nationally representative (19–21).

The present study focused on Chinese licensed doctors as the research sample. Licensed doctors refer to healthcare workers who have obtained qualifications in accordance with the law and have registered to practice in healthcare institutions. This category includes practicing doctors and practicing assistant doctors, whose health and security are legally protected (10). This study aimed to provide real and accurate information regarding the prevalence of WPV while analyzing the characteristics associated with a high occurrence of such incidents. Additionally, this study further examined licensed doctors’ perceptions of effective measures to control and prevent WPV based on the analysis of relevant characteristics. These findings may help Chinese policymakers understand licensed doctors’ urgent needs and implement effective prevention and control measures.

## 2 Materials and methods

### 2.1 Research design

A cross-sectional survey was conducted with purposive sampling method in China between January and June 2022. An online survey was used for data collection to comply with prevention and control measures during the Coronavirus disease 2019 (COVID-19) pandemic. Participants needed to meet the following eligibility criteria: a minimum of 2 years of work experience as doctors in China, possession of the relevant practicing qualification or certification, ability to use the Internet to complete the online questionnaire, and agreement to the terms and conditions for consent. All information collected during the investigations was treated as confidential and anonymous.

A total of 74,373 questionnaires were collected, and Excel 2016 were employed for questionnaire entry and data cleaning. During the process of data cleaning, 68 questionnaires were excluded for repeated submissions, incomplete submissions, or inconsistent responses. Ultimately, a total of 74,305 valid questionnaires were obtained, resulting in an effective response rate of 99.91%.

### 2.2 Measures

#### 2.2.1 Sociodemographic characteristics

This study examined the following sociodemographic characteristics: gender (male or female), age (≤25, 26–35, 36–45, 46–55, ≥56), marital status (single, married, divorced, or others), and level of education (technical secondary school and below, senior college, bachelor’s degree, master’s degree, doctoral degree, and others).

#### 2.2.2 Occupational characteristics

Occupational characteristics collected in this study included professional title (primary and below, medium, sub-senior, senior), registration type (clinical medicine, stomatology, traditional Chinese medicine, or public health), working area (provincial capital, municipality, county-level city, county town, or township), hospital type (public hospital, non-public hospital, or clinic), and hospital classification (tertiary, secondary, primary, community, or other).

#### 2.2.3 Prevalence of workplace violence and healthcare workers’ response

This study adopted the definition of WPV from International Labor Office (ILO), International Council of Nurses (ICN), World Health Organization (WHO), and Public Services International (PSI). WPV has been divided into physical and non-physical categories (22, 23).

The prevalence of WPV was evaluated through a self-reported question asking participants if they had ever experienced certain behaviors from patients or patients’ relatives. The options included physical violence, non-physical violence, both physical and non-physical violence, or neither physical nor non-physical violence.

Doctors’ perceptions of effective WPV prevention and control measures were evaluated by five options derived from the *2018 White Paper on the Occupational Status of Chinese Physicians*. These options included “intensifying crackdowns on criminal behaviors,” “improving the citizen’s level of education,” “building a defense system at healthcare institutions’ entrance,” “strengthening security education for doctors” and “truthfully reporting WPV incidents.” These measures have been proven effective in preventing and controlling WPV (24–27).

### 2.3 Statistical analysis

The sociodemographic characteristics, occupational characteristics, and prevalence of WPV are presented in numbers and percentages. The Chi-square test was used to compare these characteristics among four different groups: those who had experienced physical violence, those who had only experienced non-physical violence, those who had experienced both physical and non-physical violence, and those who had not suffered from either type of violence. Binary logistic regression with the enter method was employed to test the factors influencing WPV among doctors. Sociodemographic and occupational characteristics were analyzed as the independent variables, while whether the participants had encountered WPV was analyzed as a dichotomous variable. The Statistical Package for the Social Sciences (SPSS) (IBM Corp. Released 2019. IBM SPSS Statistics for Windows, Version 26.0. Armonk, NY: IBM Corp) was utilized for statistical analysis. All tests were two-tailed, and *p* < 0.05 was considered statistically significant.

## 3 Results

### 3.1 Sociodemographic and occupational characteristics and prevalence of WPV

Table 1 provides an overview of the sociodemographic and occupational characteristics of the study population. A total of 74,305 respondents completed the survey, with 36,267 being male (48.81%) and 38,038 being female (51.19%). More than one-third of the respondents were aged between 36 and 45. Most respondents were married (86.70%) and had obtained at least an undergraduate degree (90.81%). A substantial proportion of participants, specifically 86.19%, were clinical doctors. Many of them worked in public hospitals, accounting for 93.25% of the sample. More than half (57.16%) of the participants worked in tertiary hospitals. Most of the participants resided in provincial capitals and municipalities, comprising 28.57% and 33.12% of all participants, respectively.

**Table 1:** Sociodemographic, occupational characteristics, and prevalence of WPV.

Variables		Physical violence	Verbal violence	Both	Neither	*χ* ^2^	*p* value
		*N* (%)	*N* (%)	*N* (%)	*N* (%)		
	Overall	353 (0.48)	29985 (40.35)	3011 (4.05)	40956 (55.12)		
Gender							
Male	36,267	226 (64.02)	15,247 (50.85)	2003 (66.52)	18,791 (45.88)	601.29	<0.01
Female	38,038	127 (35.98)	14,738 (49.15)	1,008 (33.48)	22,165 (54.12)		
Age							
≤25	1,184	20 (5.67)	438 (1.46)	54 (1.79)	672 (1.64)	457.59	<0.01
26–35	23,018	137 (38.81)	10,204 (34.03)	896 (29.76)	11,781 (28.77)		
36–45	27,254	122 (34.56)	11,124 (37.10)	1,103 (36.63)	14,905 (36.39)		
46–55	18,286	56 (15.86)	6,785 (22.63)	756 (25.11)	10,689 (26.10)		
≥56	4,563	18 (5.10)	1,434 (4.78)	202 (6.71)	2,909 (7.10)		
Marital							
Single	8,112	64 (18.13)	3,569 (11.90)	365 (12.12)	4,114 (10.04)	81.96	<0.01
Married	64,420	275 (77.90)	25,714 (85.76)	2,560 (85.02)	35,871 (87.58)		
Divorced	1,558	12 (3.40)	620 (2.07)	78 (2.59)	848 (2.07)		
Others	215	2 (0.57)	82 (0.27)	8 (0.27)	123 (0.30)		
Education							
Technical Secondary School and below	1,224	7 (0.57)	251 (20.51)	44 (3.59)	922 (75.33)	428.016	<0.01
Senior College	5,601	39 (0.70)	1903 (33.98)	222 (3.96)	3,437 (61.36)		
Bachelor’s degree	46,283	233 (0.50)	18,891 (40.82)	2017 (4.36)	25,142 (54.32)		
Master’s degree	16,755	58 (0.35)	7,259 (43.32)	582 (3.47)	8,856 (52.86)		
Doctoral degree	4,442	16 (0.36)	1,681 (37.84)	146 (3.29)	2,599 (58.51)		
Professional Title							
Primary and below	1,224	142 (40.23)	7322 (24.42)	777 (25.81)	10185 (24.87)	145.84	<0.01
Medium	5,601	116 (32.86)	11399 (38.02)	1063 (35.30)	14406 (35.17)		
Sub senior	46,283	61 (17.28)	7534 (25.13)	770 (25.57)	10425 (25.45)		
Senior	16,755	34 (9.63)	3730 (12.44)	401 (13.32)	5940 (14.50)		
Registration type							
Clinical	64,045	302 (85.55)	26,082 (86.98)	2,633 (87.45)	35,028 (85.53)	79.05	<0.01
Stomatology	2,268	10 (2.83)	881 (2.94)	66 (2.19)	1,311 (3.20)		
TCM*	6,899	33 (9.35)	2,702 (9.01)	263 (8.73)	3,901 (9.52)		
PH*	1,093	8 (2.27)	320 (1.07)	49 (1.63)	716 (1.75)		
Working area							
Provincial capital	21,229	91 (25.78)	8,793 (29.32)	761 (25.27)	11,584 (28.28)	72.69	<0.01
Municipality	24,613	117 (33.14)	9,926 (33.10)	1,022 (33.94)	13,548 (33.08)		
County-level city	9,874	49 (13.88)	3,949 (13.17)	406 (13.48)	5,470 (13.36)		
County town	13,258	68 (19.26)	5,407 (18.03)	581 (19.30)	7,202 (17.58)		
Township	5,331	28 (7.93)	1910 (6.37)	241 (8.00)	3,152 (7.70)		
Hospital type							
Public hospitals	69,289	323 (91.50)	28152 (93.89)	2782 (92.39)	38032 (92.86)	154.12	<0.01
Non-public hospital	4,273	24 (6.80)	1689 (5.63)	204 (6.78)	2356 (5.75)		
Clinic	743	6 (1.70)	144 (0.48)	25 (0.83)	568 (1.39)		
Hospital classification							
Tertiary	42,473	165 (46.74)	17292 (57.67)	1610 (53.47)	23406 (57.15)	136.63	<0.01
Secondary	22,145	126 (35.69)	9071 (30.25)	987 (32.78)	11961 (29.20)		
Primary	3,660	27 (7.65)	1414 (4.72)	174 (5.78)	2045 (4.99)		
Community	5,208	22 (6.23)	1976 (6.59)	190 (6.31)	3020 (7.37)		
Others	819	13 (3.68)	232 (0.77)	50 (1.66)	524 (1.28)		

*TCM, Traditional Chinese Medical; PH, Public Health.

A total of 33,349 participants (44.88%) reported experiencing WPV in the last 12 months. Among them, 353 (1.06%) only suffered from physical violence, 29,985 (89.91%) only suffered from non-physical violence, and 3,011 (9.03%) experienced both physical violence and non-physical violence. The results indicate that the vast majority of reported WPV incidents were non-physical in nature.

### 3.2 Binary logistic regression analysis of WPV incidents

Binary logistic regression analysis suggested that gender, age, marital status, and education level were all correlated with WPV incidents (see Table 2 for details). Among them, there was a fluctuating correlation between age and experience of WPV. Compared with doctors over 56 years of age, doctors in other age groups were more likely to experience WPV and showed a negative correlation. Doctors between 26 and 35 years of age (OR = 1.778, 95CI% 1.634 to 1.935) were more likely to experience WPV. Being unmarried (OR = 1.174, 95%CI 1.111 to 1.241) was a risk factor for WPV. Additionally, there was a positive correlation between education status and WPV. This trend declined among those who had a doctoral degree, while those with a master’s degree (OR = 2.021, 95%CI 1.739 to 2.349) had a higher risk of experiencing WPV than others.

**Table 2:** Binary logistic regression analysis of WPV experience.

Variables	B	OR	95%CI	*p* value
Gender (Females *Ref.*)				
Males	0.334	1.396	(1.355,1.439)	<0.01
Age (≥56 Ref.)				
≤25	0.442	1.555	(1.342,1.803)	<0.01
26–35	0.576	1.778	(1.634,1.935)	<0.01
36–45	0.354	1.425	(1.323,1.536)	<0.01
46–55	0.210	1.234	(1.151,1.323)	<0.01
Marital (Married *Ref.*)				
Single	0.161	1.174	(1.111,1.241)	<0.01
Divorced	0.153	1.165	(1.052,1.291)	<0.01
Others	0.158	1.171	(0.890,1.541)	0.26
Education (Technical Secondary School and below *Ref.*)				
Senior College	0.436	1.547	(1.336,1.791)	<0.01
Bachelor’s degree	0.649	1.913	(1.657,2.208)	<0.01
Master’s degree	0.704	2.021	(1.739,2.349)	<0.01
Doctoral degree	0.507	1.661	(1.413,1.952)	<0.01
Professional Title(Primary and below *Ref.*)				
Medium	0.161	1.175	(1.122,1.230)	<0.01
Sub senior	0.177	1.194	(1.125,1.267)	<0.01
Senior	0.170	1.185	(1.099,1.278)	<0.01
Registration type (Public health *Ref.*)				
Clinical medicine	0.294	1.342	(1.177,1.529)	<0.01
Stomatology	0.177	1.194	(1.023,1.394)	0.02
Traditional Chinese Medicine	0.151	1.163	(1.013,1.335)	0.03
Working area (Township *Ref.*)				
Provincial capital	0.113	1.120	(1.036,1.211)	<0.01
Municipality	0.075	1.078	(0.995,1.167)	0.07
County-level city	0.058	1.060	(0.975,1.152)	0.17
County town	0.080	1.083	(0.998,1.175)	0.06
Hospital classification (Tertiary hospital *Ref.*)				
Secondary	0.097	1.102	(1.058,1.149)	<0.01
Primary	0.154	1.166	(1.075,1.266)	<0.01
Community	0.156	1.169	(1.087,1.258)	<0.01
Others	−0.041	0.960	(0.822,1.122)	0.61
Hospital type (Public hospitals *Ref.*)				
Non-public hospitals	0.017	1.007	(0.944,1.075)	0.83
Clinics	−0.545	0.580	(0.480,0.701)	<0.01
Constant	−1.908	0.148		<0.01

Occupational characteristics such as professional title, type of registration, and occupational characteristics including the location, classification, and types of hospitals where a physician worked were all related to WPV. The professional title was positively correlated with whether they had experienced WPV, such as education status and age, and when it reached the senior title, the trend was moderated. Doctors with a sub senior title (OR = 1.194, 95%CI 1.125 to 1.267) were more likely to suffer from WPV. Doctors registered in public health were less likely to experience WPV than others, which may be related to the content of their work. Doctors registered as working in clinical medicine (OR = 1.342, 95CI% 1.177 to 1.529) were the most likely to experience frequent WPV. Compared with doctors in township hospitals, doctors working in provincial capitals (OR = 1.120, 95CI% 1.036 to 1.211) were more likely to suffer from WPV, while doctors in tertiary hospitals were less likely to suffer from WPV. Additionally, doctors in primary hospitals (OR = 1.166, 95%CI 1.075 to 1.266) and community hospitals (OR = 1.169, 95%CI 1.087 to 1.258) experienced a higher frequency of WPV, while doctors in clinics (OR = 0.580, 95%CI 0.480 to 0.701) were less likely to experience WPV than doctors in public hospitals.

In China, licensed doctors are divided into four categories: clinical, stomatology, traditional Chinese medicine, and public health. Within the clinical category, doctors are further divided into various specialties, such as internal medicine, surgery, pediatrics, ophthalmology, otolaryngology, dermatology and venereology, medical imaging, medical laboratory and pathology, emergency medicine, rehabilitation medicine, preventive medicine, mental health, obstetrics and gynecology, anesthesiology, esthetic surgery, cosmetic dermatology, and general medicine. The type of doctor’s occupation is registered with the health administrative department based on their chosen department within the hospital.

The results of the binary logistic regression revealed that the type of registration had an influence on WPV, with clinicians being more susceptible to WPV than public health physicians. Therefore, further subgroup analysis was conducted to explore the factors affecting WPV among clinicians, incorporating sociodemographic and occupational characteristics into the model. Various factors, such as gender, age, marital status, education level, professional title, and occupational type, significantly influenced the exposure of clinicians to WPV, which aligned with the overall findings. Male (OR = 1.375,95%CI 1.326 to 1.426) clinicians were more susceptible to WPV. Furthermore, younger age was associated with a higher likelihood of experiencing WPV. Single (OR = 1.204, 95%CI 1.131 to 1.282) and divorced (OR = 1.160, 95%CI 1.036 to 1.299) clinicians were more prone to WPV than married physicians. Moreover, higher education levels and professional titles were correlated with an increased risk of WPV. Specifically, clinicians specializing in internal medicine (OR = 1.084, 95%CI 1.029 to 1.142), surgery (OR = 1.105, 95%CI 1.041 to 1.172), pediatrics (OR = 1.181, 95%CI 1.114 to 1.252), emergency medicine (OR = 1.915, 95%CI 1.722 to 2.129), and mental health (OR = 1.431, 95%CI 1.301 to 1.574) were more likely to encounter WPV, with emergency medicine and mental health doctors experiencing WPV most frequently, consistent with previous studies. On the other hand, clinicians in medical imaging (OR=0.738, 95%CI 0.689 to 0.789), medical laboratory and pathology (OR = 0.363, 95%CI 0.320 to 0.411), preventive medicine (OR = 0.667, 95%CI 0.534 to 0.832), obstetrics and gynecology (OR = 0.793, 95%CI 0.737 to 0.854), and anesthesiology (OR = 0.335, 95%CI 0.313 to 0.360) were less susceptible to WPV. This finding is in line with the fact that these clinicians have limited long-term contact with patients compared to other specialties (Table 3).

**Table 3:** Binary logistic regression analysis of clinicians’ WPV experience.

Variables	B	OR	95%CI	*p* value
Gender (Females *Ref.*)				
Males	0.318	1.375	(1.326,1.426)	<0.01
Age (≥56 Ref.)				
≤25	0.441	1.555	(1.314,1.840)	<0.01
26–35	0.612	1.844	(1.682,2.023)	<0.01
36–45	0.387	1.473	(1.359,1.597)	<0.01
46–55	0.259	1.295	(1.202,1.395)	<0.01
Marital (Married *Ref.*)				
Single	0.186	1.204	(1.131,1.282)	<0.01
Divorced	0.149	1.160	(1.036,1.299)	0.010
Others	0.108	1.114	(0.819,1.516)	0.491
Education (Technical Secondary School and below *Ref.*)				
Senior College	0.347	1.415	(1.195,1.677)	<0.01
Bachelor’s degree	0.517	1.677	(1.422,1.979)	<0.01
Master’s degree	0.520	1.681	(1.415,1.998)	<0.01
Doctoral degree	0.309	1.362	(1.133,1.636)	0.001
Professional Title(Primary and below *Ref.*)				
Medium	0.167	1.181	(1.121,1.244)	<0.01
Sub senior	0.151	1.164	(1.089,1.243)	<0.01
Senior	0.093	1.098	(1.010,1.192)	0.027
Working area (Township *Ref.*)				
Provincial capital	0.149	1.160	(1.058,1.272)	0.002
Municipality	0.070	1.073	(0.978,1.176)	0.136
County-level city	0.063	1.065	(0.967,1.172)	0.201
County town	0.110	1.116	(1.016,1.226)	0.021
Hospital classification (Tertiary *Ref.*)				
Secondary	0.061	1.063	(1.015,1.113)	0.009
Primary	−0.016	0.984	(0.890,1.089)	0.755
Community	−0.043	0.958	(0.866,1.060)	0.405
Others	−0.235	0.791	(0.655,0.955)	0.015
Hospital type (Public hospitals *Ref.*)				
Non-public hospitals	0.040	1.041	(0.968,1.119)	0.278
Clinics	−0.765	0.465	(0.360,0.601)	<0.01
Occupational type				
Internal medicine	0.081	1.084	(1.029,1.142)	0.002
Surgery	0.100	1.105	(1.041,1.172)	0.001
Pediatrics	0.166	1.181	(1.114,1.252)	<0.01
Eye otolaryngology	0.076	1.079	(0.973,1.196)	0.148
Dermatology and venereology	0.069	1.071	(0.927,1.239)	0.351
Medical Imageology	−0.304	0.738	(0.689,0.789)	<0.01
Medical laboratory and pathology	−1.013	0.363	(0.320,0.411)	<0.01
Emergency medicine	0.649	1.915	(1.722,2.129)	<0.01
Rehabilitation medicine	−0.123	0.885	(0.749,1.044)	0.147
Preventive medicine	−0.405	0.667	(0.534,0.832)	<0.01
Mental health	0.359	1.431	(1.301,1.574)	<0.01
Obstetrics and gynecology	−0.232	0.793	(0.737,0.854)	<0.01
Anesthesiology	−1.092	0.335	(0.313,0.360)	<0.01
esthetic surgery	0.186	1.204	(0.870,1.667)	0.263
Cosmetic dermatology	0.144	1.155	(0.804,1.660)	0.436
General medicine	0.005	1.005	(0.927,1.090)	0.900
Others	−0.041	0.960	(0.866,1.065)	0.442
Constant	−1.373	0.253		<0.01

### 3.3 Prevention and control measures

Table 4 presents licensed doctors’ perceptions of effective measures to prevent and control WPV. The top three effective measures were as follows: intensifying crackdowns on criminal behaviors (*n* = 32,870, 44.24%), improving the general public’s level of education (*n* = 15,756, 21.20%), and increasing security measures at the entrances of healthcare institutions (*n* = 13,549, 18.23%). Less than 10% of licensed doctors considered strengthening security education for healthcare workers (*n* = 5697, 7.67%) and truthfully reporting WPV incidents (*n* = 6433, 8.66%) to be effective in preventing and controlling WPV.

**Table 4:** Prevention and control measure.

Measures	2022	
	*n*	%
Intensifying crackdowns on criminal behaviors	32,870	44.24
Improving the citizen’s level of education	15,756	21.20
Building a defense system at healthcare institutions’ entrance	13,549	18.23
Truthfully reporting WPV incidents	6,433	8.66
Strengthening security education for doctors	5,697	7.67

## 4 Discussion

This study examined the influencing factors and response measures to WPV in China. This is the first nationwide study to investigate WPV after the introduction of relevant laws and policies. There were several critical findings on the prevalence of WPV against doctors, including associated influencing factors and preventive and control measures. First, less than half of doctors (44.88%) had experienced WPV in the past year. Second, most WPV incidents in China were non-physical in nature. Third, WPV was associated with certain sociodemographic characteristics represented by sex, age, and education. Fourth, WPV was associated with work-related factors, such as type of clinical registration, location of the hospital, and whether the doctors worked in public, private or community hospitals. Last, doctors reported that intensifying crackdowns on criminal behavior was an effective measure to prevent and control WPV.

Based on previous studies, the rate of WPV was reported to range from 45 to 80% (27–29). In this study, the prevalence of WPV was 44.88%, which was lower than that in previous studies. The results also revealed that the prevalence of WPV was close to that of foreign countries, such as Italy, the United States, and the United Kingdom (30–34). In addition, the prevalence of physical violence among doctors has dramatically decreased, which may account for the introduction of various laws and policies. Non-physical violence has remained the most common type, which indicates a future direction of policymaking and legislation for Chinese authorities.

The causes of WPV against doctors are very complex. In terms of gender, the outcome of this study is like those of numerous prior investigations, indicating that male doctors are more likely to encounter WPV than their female counterparts (26, 28, 35, 36). Previous studies have also identified age as a significant influencing factor for WPV in China (26). This study found that doctors aged 26 to 35 or younger tended to have an increased likelihood of experiencing WPV owing to insufficient experience communicating with patients (37). Regarding the level of education, previous research has indicated that doctors who hold a master’s degree are more likely to experience WPV (28). This study also found that highly educated doctors (who held a master’s degree or above) suffered a high percentage of WPV.

The results also revealed some interesting findings. The risk of experiencing WPV was much higher for clinically registered doctors than for other types of registered doctors. The reason may be that clinical doctors are required to provide medical services and publish papers, which results in a heavy workload and psychological pressure (3, 24, 38). At the same time, the analysis of clinicians’ experience with WPV showed that doctors specializing in internal medicine, surgery, pediatrics, emergency medicine, and mental health were more likely to encounter WPV, with emergency medicine and mental health doctors experiencing WPV most frequently, which is in accordance with previous studies (39–42). Doctors working in medical imaging, medical examination and pathology, preventive medicine, obstetrics and gynecology, and anesthesiology were less likely to be affected by WPV. Most of the horrific incidents of killing and injuring doctors in China (represented by the Civil Aviation General Hospital incident) in recent years have occurred in the emergency department and surgical department. This phenomenon can be explained by the intolerable pain of patients, extremely long waiting times, poor security and overcrowded surroundings (23). For the hospital, the medical treatment process should be optimized to reduce the waiting time of patients. Moreover, healthcare administrative departments should continue to prioritize hierarchical medical systems and reduce the burden of doctors in tertiary hospitals. In addition to hospitals and health authorities, another study showed that social media also plays a key role in promoting occupational safety for physicians (3). This study also noted that WPV incidents mostly occurred in cities, which is consistent with a previous study (24). In China, tertiary and secondary hospitals are mainly responsible for specialized care, while primary-level hospitals are expected to provide basic medical services as well as preventive care (43). A previous study showed that doctors working in tertiary hospitals were more likely to be exposed to WPV during the previous 12 months. Compared with previous studies, this study found that community hospital doctors were vulnerable to WPV. The prevalence of WPV in community hospitals may be ascribed to the unrealistic expectations and distrust (44).

This study found that there was not much difference in Chinese doctors’ perceptions of effective interventions for WPV. With a larger national sample of licensed doctors, a higher rate of licensed doctors reporting that intensifying crackdowns on criminal behaviors was the most effective measure to prevent WPV against them. This is in line with the Chinese Medical Doctor Association’s call for “zero tolerance of violence,” but in practice, attention should still be paid that a law-based and evidence-based process for medical disputes should be established (45). In addition, increasing attention has been focused on building a defense system, suggesting that doctors perceived relevant security measures were insufficient concerning the number of security personnel, quality and stability of the security team, and security measures. The proportion of licensed doctors who believed that truthfully reporting WPV incidents would help prevent and control WPV remained lowest. This is a substantial decline compared with the survey conducted with Chinese licensed doctors in 2011 (53.75%) and 2014 (84.31%), which indicates that in recent years, the news authorities have been more rigorous in reporting medical cases, and the impartiality has been gradually affirmed by doctors, and the media has played a relatively positive role in which is in accordance with the previous study (3, 27). The results also showed that, “improving the citizen’s level of education” and “increasing doctors’ self-protection awareness” are still what doctors consider effective measures to control WPV. Therefore, public education should be strengthened to reduce patients’ unrealistic expectations of medical measures and improve their cognition. Meanwhile, it is very necessary to carry out self-protection training for doctors (23, 46, 47).

## 5 Strengthen and limitations

Our findings can provide guidance for policymaking related to the prevention and control of WPV against doctors. The results of this study emphasize the importance of implementing proactive measures and introducing legal provisions to mitigate the risk of WPV in medical settings. Additionally, our findings suggest an urgent need for prevention strategies, particularly in high-risk departments such as internal medicine, surgery, pediatrics, emergency medicine, and mental health, where the incidence of WPV is higher than in other departments. In the future, China can further enhance healthcare worker training, improve hospital infrastructure, and implement specific measures to protect doctors from WPV in these key departments.

This study has several limitations. First, the present study involved only licensed doctors, who are regular workers and a high-risk WPV group, since they undertake clinical work and deal with patients for a prolonged period. Second, the effects of the COVID-19 pandemic on the prevalence of WPV cannot be ignored. During the pandemic, the heavy workload, stressful work environment, and insufficient access to medical resources significantly impacted the mental health of doctors, resulting in a notable increase in violence toward doctors (39–41). Third, this was a retrospective study, and the data were collected in a self-reported manner, which might have led to cognitive bias.

## 6 Conclusion

Workplace violence against healthcare workers in China poses a serious threat to the well-being of doctors. The results of this cross-sectional study suggest that the estimated prevalence of WPV against licensed doctors in China has slightly decreased, and sociodemographic characteristics and occupational characteristics are all risk factors. Overall, age and hospital classification are the negative factors affecting the prevalence of WPV, while education, professional title and the administrative level where the hospital is located are the positive factors. Male clinicians, who were single, with master’s degree and professional title were subsenior were most likely to occur WPV. Furthermore, WPV occurred mostly in provincial capitals, public hospitals, primary and community hospitals, and departments of internal medicine, surgery, pediatrics, emergency medicine and mental health. In terms of doctors’ attitudes about how to reduce the incidence of WPV, 44.24% of doctors perceived that strengthening crackdowns on criminal behaviors was the most effective measure to prevent WPV against healthcare staff. However, non-physical forms of WPV remain prevalent. This study can further serve as a guide for policymakers to implement effective measures to control and prevent WPV while creating a more harmonized working environment for licensed doctors in China.

## Data availability statement

The raw data supporting the conclusions of this article will be made available by the authors, without any undue reservation. Enquiries to access these datasets can be directed to the corresponding authors.

## Ethics statement

This research was approved by the Institutional Review Board of Peking University (No. IRB00001052-22053). The patients/participants provided their written informed consent to participate in this study.

## Author contributions

YW, JX, and WC: conceptualization. JS: data curation and project administration. WC: writing-original draft. JX, YW, WC, JS, and YbW: writing-reviewing and editing. All authors contributed to the article and approved the submitted version.
